# Successful Suture of Traumatic Rupture of Spleen

**Published:** 1916-05

**Authors:** 


**Successful Suture of Traumatic Rupture of Spleen.** Joseph J. Levin, Boksburg Hospital, Med. Jour. of South Africa, Dec., 1915, reports the case of a native aged 18, who had been kicked in the back and struck with the fist in the abdomen by his master. He was first seen the following day, showing the ordinary signs of shock and internal haemorrhage. The liver was examined after section and found intact. A 2-inch tear of the capsule of the spleen was found, and a large amount of dark blood was evacuated from the peritoneal cavity. Recovery followed upon suture. Michelson is quoted as giving a mortality of 33.2% in a series of 298 cases.

